# Rebels? No, Simply Scientists

**DOI:** 10.1371/journal.pbio.0060242

**Published:** 2008-09-30

**Authors:** Michel Morange

## Abstract

Michel Morange reviews *Rebels*, *Mavericks*, *and Heretics in Biology*, an alternative history of modern biological thought.

The problem of creativity is common to the arts and sciences. What distinguishes geniuses from ordinary mortals? In the arts, from Mozart to van Gogh, creativity has frequently been associated with the artist's opposition to the society of their time. A good artist is a rebel. Paradoxically, whereas science might appear as a progressive rational construction of new knowledge, the same relation has been postulated between rebellion and scientific creativity. There are many historical accounts of how scientists who made decisive breakthroughs saw their ideas rejected, and became “rebels.”

*Rebels, Mavericks, and Heretics in Biology* presents a collection of essays by different authors on biologists who were, in one way or another, considered rebels [1]. What do they have in common? Is it possible to find biographical clues to the forging of this spirit of rebellion?

This book can be appreciated from three different points of view. The first is simply to consider it as a rich collection of studies of scientists who played a significant, although sometimes marginal, role in the development of the life sciences in the 20th century. The contributions of some of them have already been studied, but there are new figures sketched here such as Carl Woese, who discovered a third branch of life, Motoo Kimura, who radically modified our vision of evolution, and Raymond Arthur Dart, who dramatically revised the scenarios on the origin of modern humans. The originality of the book is also in the comparable size and format of the presentations. A good balance has been reached between a short biographical introduction and a longer presentation of the original work accomplished and the obstacles and opposition encountered. The authors of these short essays have diverse training and skills, but the quite rigid organization of the book facilitates comparison of the different chapters.

**Figure:**
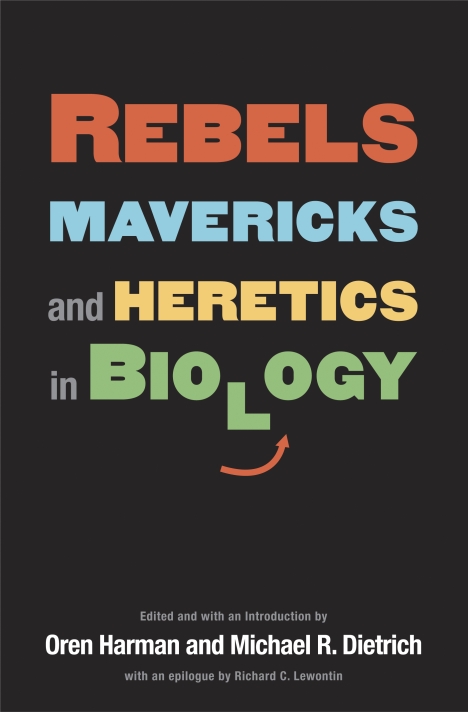


One great merit of this book is to have included figures from diverse biological disciplines, ranging from molecular to evolutionary biology, ecology, and neurophysiology. Such diversity is rare and gives this book a particular flavor. The quality and importance of the authors (among them Garland Allen, David Hull, and Michael Ruse) is another richness, which should convince all those interested in the development of the life sciences over the last century to buy this book and read and browse at their own pace.

But the ambitions of the editors of this collective work were higher. The aim was to write the history of 20th century biology from the perspectives of the rebels, the mavericks, and the heretics. There is a strong trend in present-day historical studies to give a voice to the “small man,” to those who go unheard, to those who lost out. By adopting such an approach, one can hope not only to obtain a different, renewed historical vision, but also to create a sort of “counterfactual” history likely to allow testing of the hypotheses that have been produced to explain such historical developments. Consider, for instance, the highly different view that Erwin Chargaff provided of the discovery of the double helix [2] in comparison with that proposed by Jim Watson [3]. Chargaff emphasized the huge role that biochemical work played in the progressive description of the DNA molecule, a role largely ignored by Jim Watson.

The second ambition of this book was to draw the characteristics of the special class of scientists called “rebels” or “mavericks.” As most of these rebels produced important results, it is a way to question the relations between rebellion and creativity in science. Are there more rebels in biology than in other sciences? This book is clearly not intended to provide an answer. But if one of the ways leading to rebellion is “focusing on exceptions” instead of “focusing on rules” (p. 11), the biological sciences, with their wealth of exceptions, could be favorable ground.

Does this book achieve its ambitions? In their introduction, Oren Harman and Michael Dietrich raise serious doubts about the possibility of defining a category of “rebels” or “heretics” in science. As they say, “the category of rebel may be elusive and nebulous” (p. 3). One should add that the words used to describe the figures portrayed in this book frequently seem excessive. In many instances, they might have been advantageously replaced simply by “original,” without any loss of meaning. Many of the people considered in this book were recipients of the Nobel Prize or other distinctions, and it is somehow difficult to consider them as “mavericks” and “heretics.” When Harman and Dietrich say that “first the maverick must choose an important and relevant problem” and “if one single thing unites all the characters featured in this book, it is that they all exhibited stubbornness and steadfastness in their challenges to orthodox thought” (p. 7), I have the feeling that this is a simple retrospective judgment, and that otherwise the names of these people would never have been in our memories: this is not an explanation of their important contributions.

The use of “iconoclast” works better. Clearly, these scientists exchanged previous methodologies and concepts for new ones, and did new experiments or established a bridge between disciplines hitherto separated. Such, for instance, was the case of Peter Mitchell, described by John Prebble and Bruce Weber, who succeeded in solving the problem of cell respiration, thus far considered as a purely biochemical problem, by importing models from membrane physiology. But in so doing, were they so different from “normal scientists”? Normal scientists would have been included in an official history of the life sciences, but such a history would not differ from the history presented in this book. As Harman and Dietrich admit, a taxonomy of rebels and iconoclasts is impossible, and I would add that if it existed, it would not differ from the taxonomy of scientists in general!

I suppose that one of the difficulties stems from the different values that one can ascribe to a rebel. One can choose a neutral point of view—e.g., they were right or wrong and it doesn't matter which. One can be sympathetic to them, believing that they were treated unfairly or supporting rebellion against the system, whatever it is. Or, in contrast, one can simply consider that they were wrong. The editors and authors have not clearly chosen between these three different attitudes, or more precisely they have not renounced a certain sympathy for these originals. Such an attitude makes the picture even fuzzier.

If this book falls short of its main objective, its major interest probably lies elsewhere: in underlining the naïve vision we have of what a rebel in science is, and more generally of how scientific knowledge is constructed. The first error is to conflate “rebels in science” with “rebels in general” or “rebels within society.” Some of the contributors come close to adopting such a naïve vision, but most chapters show that such an identification has no sense. The reason is that to be a heretic or a rebel is generally not in the nature of the character, but in the historical circumstances that prevented a particular discovery or model from being adopted. The nature and strength of the orthodoxies they opposed and their reasons for fighting them were highly diverse too. Obstacles may have their roots in racial or social prejudices—as when Dart proposed that modern humans originated in Africa; in the solidity of the model that is challenged—as when Richard Goldschmidt opposed the corpuscular nature of the gene; or in the weakness of the challenging theory, either because experiments were inconclusive, as in the case of Howard Temin, or because the results could not be related to previous knowledge: such is the case studied by Ute Deichmann when Oswald Avery suggested that the genetic material was DNA. How was it possible to attribute a genetic function to a molecule with a supposed “monotonous” structure, and without known chemical relations with the characters they apparently controlled?

Many of the scientists described in this book considered themselves rebels, but in most cases this was a retrospective self-description, a way to valorize their own scientific path. In some cases, such as Stephen Jay Gould, being a rebel even appears to be a strategy to attain and keep power. It can be a dangerous strategy when these scientists try to replay the game, and consider that, since they are rebels, all their contributions and results are original and deserve to be discussed. To be where people do not expect you is obviously a good strategy on the battlefield—not always in science.

In addition, scientists rarely have the same vision of their originality as the rest of the scientific community. Nathaniel Comfort shows that Barbara McClintock considered her major contribution to be the discovery of a new mechanism of gene regulation, whereas she was applauded for her discovery of transposition. This is an easily explainable confusion between the personal feeling of what was the most difficult obstacle to overcome, and the historical judgment that considers merit in terms of the scientific developments this discovery generated.

Originality and novelty can be at the origin of a research project, but they can also suddenly appear at another stage during the project's development. Ute Deichmann shows that the work of Oswald Avery was well planned, but the result he obtained in Pneumococcus—that the “transforming principle,” the substance that induces predictable and heritable changes, is made of DNA—was not. Scientists would have expected to find carbohydrates, the component of the cellular structure that is modified during transformation, or proteins, considered by most biologists at that time as the major component of the genetic material—not DNA, whose function was totally unknown. In the case of Carl Woese, Jan Sapp reminds us that his scientific project was quite original—to understand how the protein synthesis machinery emerged during evolution by working on the most “primitive” bacteria. But the “true” revolution he introduced was the discovery of a third branch in the living kingdom—the archaea; the first project being still in its infancy. Rebels in science are not born rebels; in the same way, revolutionary transformations in science emerge abruptly from scientific developments: they are not already in the lines of research that generated them. Let us definitively abandon this recurrent preformist vision: the history of science is history, with all its contingencies. Neither the roles nor the words of the play are prewritten. This is clearly demonstrated by the diversity of the scientific trajectories sketched in this book.

Nor can the direction of scientific developments be anticipated. Harman and Dietrich remark that, paradoxically, to be revolutionary in science may mean to come back to ideas and models of the past. The model of a political system that the supporters of the French revolution had in mind was ancient Greece and Rome! To consider a transformation as revolutionary or not depends upon the tempo one adopts, whether the historical study is focused on short or long periods of time.

These biographical sketches also demonstrate that scientific developments are dependent on, but not constrained by, the “controlling body of peers” described by Richard Lewontin in his epilogue. Every scientist can acquire a standing that allows him to take some risks, to explore new paths without losing the consideration of his scientific peers. New results or approaches that cannot be related to previous ones are rejected, but not forgotten. They are ready to re-emerge, as in the case of the discovery of reverse transcription by Howard Temin. A way to relate the new observations on oncogenic RNA viruses to previous knowledge had been discovered: in this case a molecular mechanism, with the discovery of reverse transcriptase.

So this book is important, not for its initial objective, but for what it affords us: a rich description of scientists and discoveries in different biological sciences. It is the best possible weapon to oppose simplistic models of science construction. We need more such beautiful and careful studies. I expect to read a second volume soon! Harman and Dietrich draw a shortlist of biologists who might have been included in this book, but were not, mainly because of the lack of a scholar able to produce a well-informed study in a limited amount of time. I would like to suggest other interesting figures, such as Boris Ephrussi, Willi Hennig, Conrad Waddington, Nikolai Timofeeff-Ressovsky, and François Jacob. The list is long, in particular if the project does not limit itself to Anglo-American culture as it has so far. After all, the drive to understand the world is a human trait that knows no national borders. And one could argue that today more than ever biologists must bridge boundaries and cultures in international collaborations that tackle increasingly difficult global challenges, such as the effects of climate change on emerging diseases, biodiversity, and life itself. It's likely that the “normal scientists” who make headway against these challenges will provide budding historians with rich tales to tell.
